# Bayesian causal inference unifies perceptual and neuronal processing of center-surround motion in area MT

**DOI:** 10.1101/2025.09.17.676722

**Published:** 2026-10-24

**Authors:** Gabor Lengyel, Sabyasachi Shivkumar, Gregory C. DeAngelis, Ralf M. Haefner

## Abstract

Center–surround interactions are a hallmark of visual processing and are especially prominent in area MT, where surround motion can either suppress or facilitate neuronal responses depending on context. However, existing mechanistic descriptions, including divisive normalization, do not explain the full diversity of these effects or their relationship to motion perception. Here, we show that both perceptual and neuronal center–surround phenomena can be understood as consequences of Bayesian causal inference over reference frames. Building on a normative model of motion perception, we derived predictions for the mean responses and variability of single MT neurons across the full four-dimensional space of center and surround directions and speeds. The model generates structured patterns of suppression, facilitation, and coordinate-frame selectivity that qualitatively match the diversity of center–surround effects reported in primate MT. Our results provide a unified computational account linking motion integration and segmentation in perception with contextual response modulation in MT, and yield testable predictions for how the visual system infers and represents reference frames.

